# Supplementary material for: Long Term Survival of Pathological Thoracolumbar Fractures Treated with Vertebroplasty: Analysis Using a Nationwide Insurance Claim Database
Source: J Clin Med. 2019 Dec 27;9(1):78. doi: 10.3390/jcm9010078 (PMC7019827; doi:10.3390/jcm9010078)
Supplement: Supplementary file 1 [file jcm-09-00078-s001.pdf]

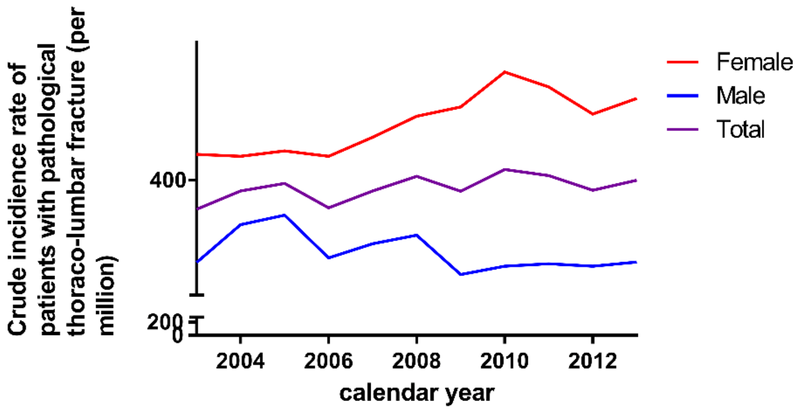

Figure S1: Crude incidence rates of patients with pathological thoracolumbar fractures during the period of 2003 to 2013 in Taiwan.

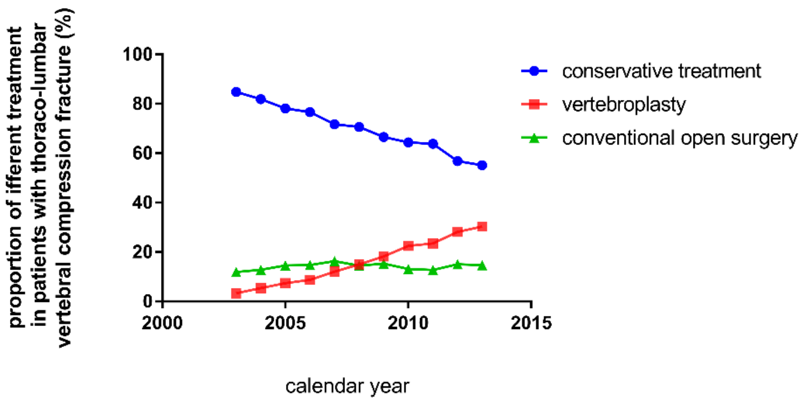

Figure S2: The proportion of patients with thoracolumbar fractures who received different treatment: (a) conservative treatment (b) vertebroplasty (c) conventional open surgery.

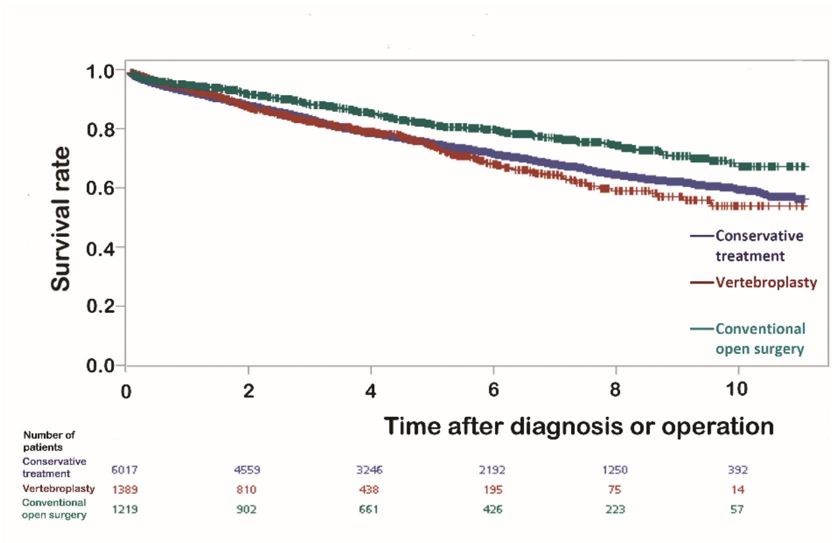

Figure S3: Kaplan–Meier survival curves of different treatment.

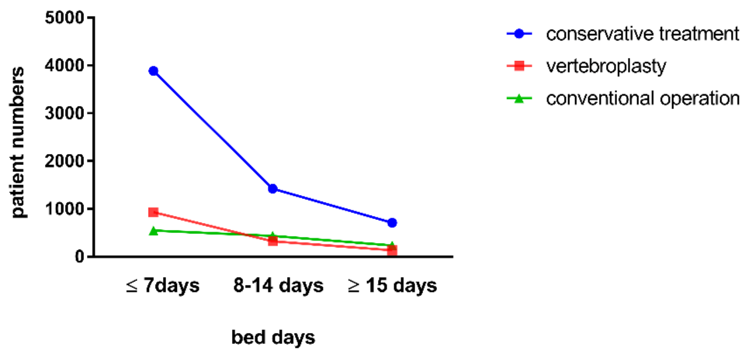

Figure 4a

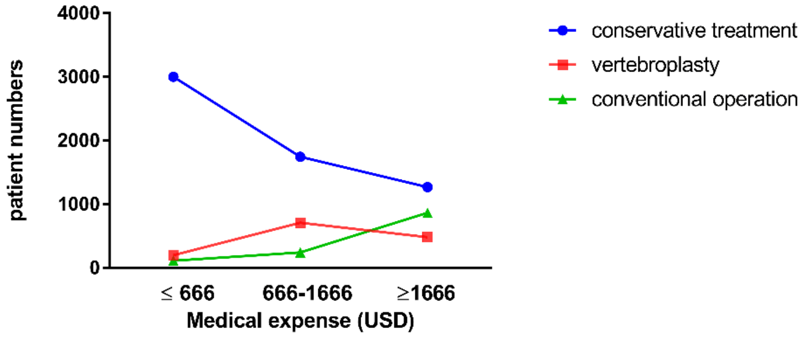

Figure 4b

Figure S4: a) Days of hospitalized of different treatments. (b) Medical expenses of different treatments during hospitalization.
